# Primary adenocarcinoma of the appendix in a child: A case report

**DOI:** 10.1186/s40792-018-0514-4

**Published:** 2018-09-04

**Authors:** Toshiaki Takahashi, Hiroshi Nouso, Masaya Yamoto, Koji Fukumoto, Naoto Urushihara

**Affiliations:** Department of Pediatric Surgery, Shizuoka Children’s Hospital, 860 Urushiyama, Aoi-ku, Shizuoka City, Shizuoka Prefecture 420-8660 Japan

**Keywords:** Primary adenocarcinoma, Appendix, Children

## Abstract

**Background:**

Primary adenocarcinoma of the appendix is a rare disease in clinical practice. Moreover, primary adenocarcinoma of the appendix in the pediatric age group is even rarer with very little cases being published. Here, we report a case of primary adenocarcinoma of the appendix with local invasion into adjacent organs in a child who was initially diagnosed as having an acute appendicitis.

**Case presentation:**

A 13-year-old girl presented with abdominal pain of 3-month duration. Imaging study showed a mass including the fecalith that occupied her pelvic and right lower abdominal cavity. Drainage of the abscess and appendectomy were performed by the preoperative diagnosis of an acute appendicitis with an appendiceal mass. Postoperative histopathological examinations revealed the appendiceal adenocarcinoma. She then received the whole mass resection, ileocecal resection with lymph node dissection. The masses were tightly adherent with infiltration into the sigmoid colon, uterus, and right ovary. These organs were all dissected, and subsequent sigmoid colostomy was performed. We preserved the left ovary for her fertility. The pathological findings demonstrated negative margins and no lymph node invasions, and final pathological stage was pT4(SI)N0M0, stage II. After the operation, she received the chemotherapy with 6 cycles of 5-fluorouracil (5-FU), leucovorin (LV), and oxaliplatin (mFOLFOX6) and subsequent 6 cycles of simplified LV and 5-FU (sLV5FU2). The patient is doing well till today on follow-up without progression of the disease 5 years after the operation.

**Conclusion:**

Primary adenocarcinoma of the appendix is exceedingly rare in children. In this report, we described one of the youngest primary adenocarcinomas of an appendix case ever reported. When encountering atypical cases of the appendicitis, we should consider the possibility of primary adenocarcinoma of the appendix as it has an extremely poor prognosis and is usually diagnosed in advanced stages.

## Background

Appendiceal cancer is a rare malignancy with an incidence of around 0.1 in 1,000,000, making up only 0.5% of all gastrointestinal malignancies [1]. The tumor itself is not aggressive but has the potential for rupture and spread throughout the peritoneum, which carries a poor prognosis [2]. This cancer is most often diagnosed following appendectomy for suspected acute appendicitis [3].

In the pediatric aged group, the incidence of colorectal cancer is rare compared with that in adults. Although early diagnosis and improved management have resulted in reduced mortality since 1980s [4], the treatment protocol of colorectal cancer in children is still controversial due to very little cases being published.

Here, we report a case of primary adenocarcinoma of the appendix (PAA) with local invasion into adjacent organs in a child who was initially diagnosed as having an acute appendicitis.

## Case presentation

### A patient

**Fig. 1:**
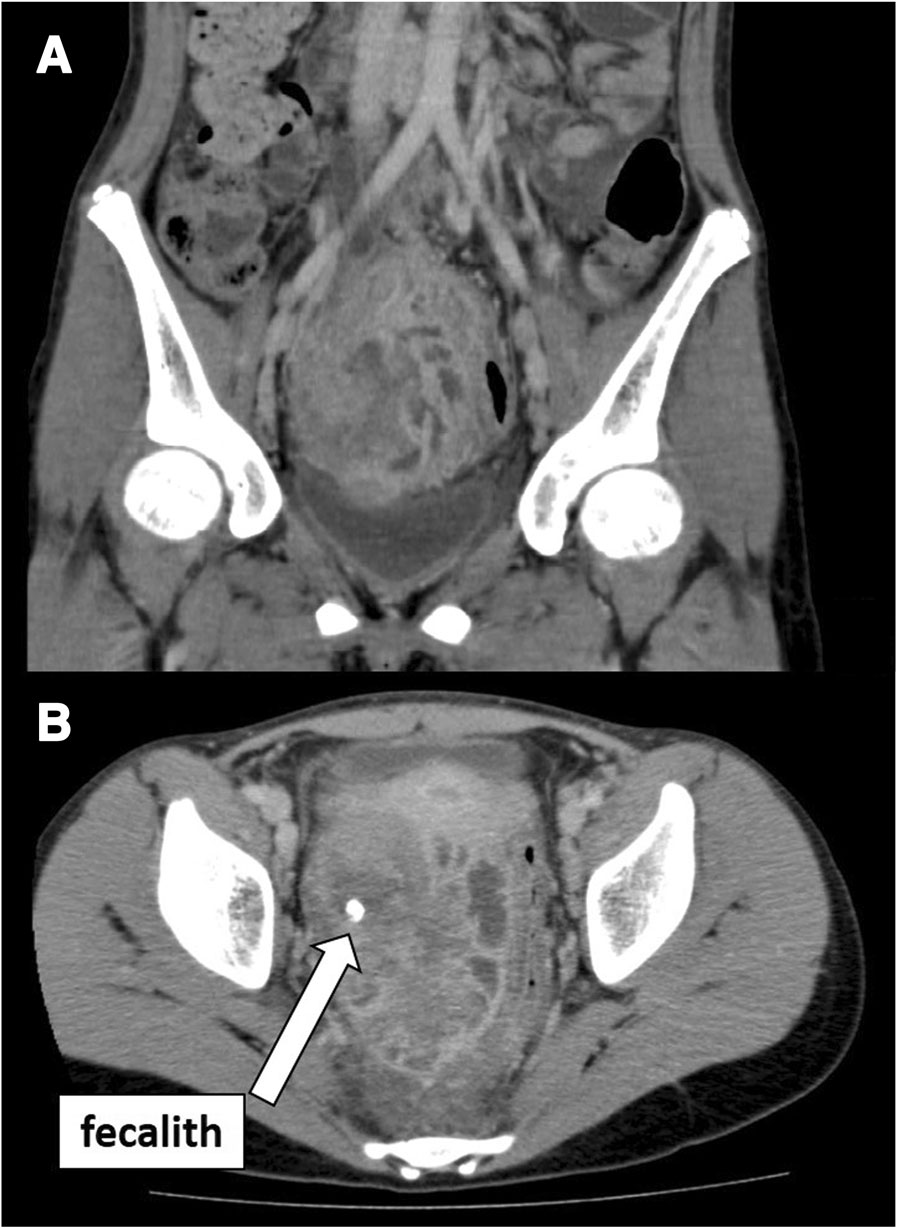
Imaging study showed a mass including the fecalith that occupied her pelvic and right lower abdominal cavity (**a** coronal, **b** horizontal)

**Fig. 2:**
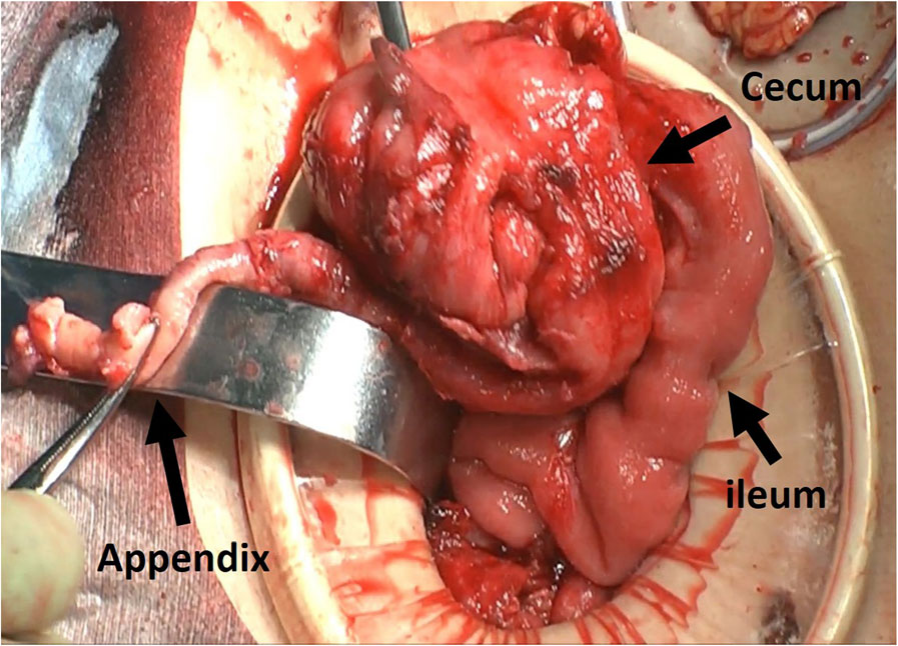
Intraoperative finding: Drainage of the abscess and appendectomy were performed by the preoperative diagnosis of an acute appendicitis with an appendiceal mass. Although the body of the appendix looked intact as well as the ileum and the cecum, the histopathological examination revealed PAA in the tip of the resected appendix

A 13-year-old girl presented with abdominal pain and bloody stool. For 3 months, she had repeated appearance of the abdominal pain and spontaneous disappearance of the symptom. At the beginning, the symptom was not strong and she had not seen a doctor. Her abdominal pain gradually got worse, and she determined to come to our hospital after she often had bloody stool. Imaging study showed a mass including the fecalith that occupied her pelvic and right lower abdominal cavity (Fig. 1). Drainage of the abscess and appendectomy were performed by the preoperative diagnosis of an acute appendicitis with an appendiceal mass (Fig. 2). Although the body of the appendix looked intact as well as the ileum and the cecum, the histopathological examination revealed PAA in the tip of the resected appendix. A radical operation for the residual tumor of PAA in the pelvic cavity was planned.

### Surgery

**Fig. 3:**
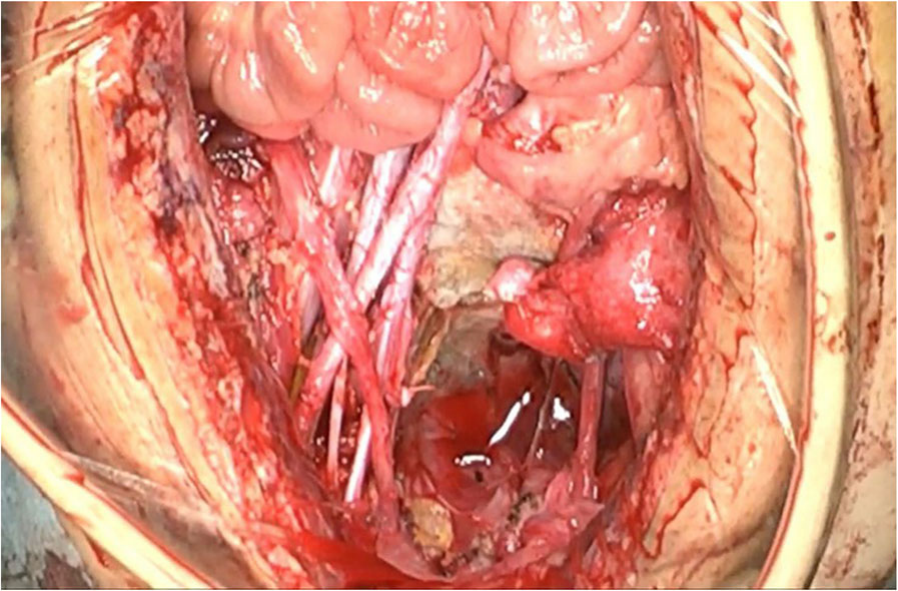
Intraoperative finding: The patient received the whole mass resection, ileocecal resection with lymph node dissection, and subsequent sigmoidectomy and adnexectomy

She then received the whole mass resection, ileocecal resection with lymph node dissection. At the first operation, we speculate that we resected body of the appendix but left the most of the mass and the tip of the appendix. Therefore, the mass which we resected in the second operation was derived from the tip of the appendix. The masses were tightly adherent with infiltration into the sigmoid colon, uterus, and right ovary. These organs were all dissected, and subsequent sigmoid colostomy was performed (Fig. 3). We preserved the left ovary for her fertility. The pathological findings demonstrated negative margins and no lymph node invasions, and final pathological stage was pT4(SI)N0M0, stage II (TNM-7th edition 2009). Postoperative course was uneventful.

### Postoperative treatment

After the operation, she received the chemotherapy with 6 cycles of 5-fluorouracil (5-FU), leucovorin (LV), and oxaliplatin (mFOLFOX6) and subsequent 6 cycles of simplified LV and 5-FU (sLV5FU2). The patient is doing well till today on follow-up without progression of the disease 5 years after the operation. We performed the stoma closure operation 2 years after the tumor resection.

### Discussion

Primary adenocarcinoma of the appendix (PAA) is an extremely rare disease in clinical practice [4]. There are no established risk factors for the development of appendix cancer. Malignant appendix tumors most often present with acute appendicitis and are diagnosed incidentally at histologic assessment of the surgical specimen. Appendix cancers may also be asymptomatic and be found incidentally. When symptoms are present, the disease process is often advanced [5].

Colorectal carcinoma in children, although rarely discovered, comprises approximately 1% of pediatric neoplasm [6]. It is also the most common primary gastrointestinal malignancy in children. However, due to the low awareness of the disease, diagnosis is usually delayed until the disease is in the advanced stage, causing prognosis to be extremely poor compared with that of adults [7].

In addition, there is no consensus for the goal of the operation for PAA in children. Generally, functional preservation operation has often been emphasized in pediatric surgery. Therefore, it is not easy to decide the grade of the extension in the radical operation for colorectal cancer such as PAA in children as there is very little reports being published. Furthermore, we have no guideline about the postoperative treatment protocol such as the resume of the chemotherapy.

We reviewed some recent literature which has been published since 2000 and found seven cases of the colorectal adenocarcinoma in children who is under 15 years old (Table 1). The tumors originate from the ascending colon in two cases, transverse colon in one case, descending colon in two cases, and rectum in two cases. Most of the cases received adjuvant chemotherapy. Although three cases had good postoperative courses, the patients in four cases died after the radical operation. The poor prognosis of the primary colorectal carcinoma in children has been re-recognized by this review [8–12].

**Table 1:** The reports of primary colorectal carcinoma in children since 2000

Case	Age (years)	Gender	Origin	Pathological results	Operation	Additional treatment	Outcome
1 [8]	13	M	A-colon	Well diff. adenocarcinoma	R-hemicolectomy	–	No recurrence
2 [9]	12	F	Rectum	Undiff. mucinous adenocarcinoma	–	Radiotherapy	Die
3 [10]	13	Unknown	D-colon	Adenocarcinoma with multiple metastasis	L-hemicolectomy and splenectomys	Chemotherapy	Die
4 [11]	9	M	Rectum	Mucinous adenocarcinoma	segmental resection	Chemotherapy	No recurrence
5 [11]	12	F	D-colon	Poorly diff. mucinous adenocarcinoma	resection of local tumor	Chemotherapy	No recurrence
6 [12]	13	M	A-colon	Poorly diff. mucinous adenocarcinoma	R-hemicolectomy	Chemotherapy	Die
7 [12]	15	M	T-colon	Mucinous adenocarcinoma	Ileosigmoidostomy bypass	Chemotherapy	Die

*M* male, *F* female, *A-colon* ascending colon, *D-colon* descending colon, *T-colon* transverse colon, *diff.* differentiated, *R-hemicolectomy* right hemicolectomy, *L-hemicolectomy* left hemicolectomy

Furthermore, Table 2 shows the reports of primary adenocarcinoma of the appendix in the recent 10 years [13–20]. The mean age at presentation for PAA is about 50 years. There is no sex predominance. Most PAAs are well differentiated, are slowly growing, and have pushing rather than infiltrating margin. The traditional treatment is right hemicolectomy. However, in the case of intra-abdominal metastasis, the treatment consists of aggressive debulking followed by chemo-radiotherapy along with it.

**Table 2:** The reports of primary adenocarcinoma of the appendix in recent 10 years

Case	Age (years)	Gender	Pathological results	Operation	Additional treatment	Outcome
1 [13]	49	M	Well diff. mucinous adenocarcinoma	Tumor resection	Chemotherapy	No recurrence for 15 months
2 [14]	57	M	Mucinous adenocarcinoma	R-hemicolectomy	Chemotherapy	Not mentioned
3 [15]	75	F	Adenocarcinoma arising focally within an appendiceal tubulovillous adenoma	Resection of the sigmoid colon, proximal rectum, and appendix	Not mentioned	Not mentioned
4 [16]	63	F	Mucinous adenocarcinoma	Ileocecal resection	–	No recurrence for 5 years
5 [17]	42	M	Low-moderate diff. mucinous adenocarcinoma	Resection of local tumor and partial bladder	Chemotherapy	No recurrence for 6 months
6 [18]	35	F	Well diff.mucinous adenocarcinoma	R-hemicolectomy	Chemotherapy	No recurrence
7 [19]	80	M	Signet ring cell adenocarcinoma	R-hemicolectomy	–	No recurrence
8 [20]	40	F	Mucinous adenocarcinoma	R-hemicolectomy	–	Not mentioned

*M* male, *F* female, *diff.* differentiated, *R-hemicolectomy* right hemicolectomy

In this report, we demonstrate a case of PAA with local invasion into adjacent organs in a child who was initially diagnosed as having an acute appendicitis. We performed radical operation and adjuvant chemotherapy. The patient is doing well without progression of the disease after 5 years. Although little has been understood about PAA, we believe our experience may provide new insights into the guideline of the standard treatment for PAA in children.

## Conclusions

Primary adenocarcinoma of the appendix is exceedingly rare in children. In this report, we described one of the youngest primary adenocarcinoma of appendix case ever reported. When encountering atypical cases of the appendicitis, we should consider the possibility of primary adenocarcinoma of the appendix as it has an extremely poor prognosis and is usually diagnosed in advanced stages.

## Abbreviations

5-FU: 5-Fluorouracil
LV: Leucovorin
PAA: Primary adenocarcinoma of the appendix
